# Metabolic flux analysis: a comprehensive review on sample preparation, analytical techniques, data analysis, computational modelling, and main application areas

**DOI:** 10.1039/d2ra03326g

**Published:** 2022-09-07

**Authors:** Bruna de Falco, Francesco Giannino, Fabrizio Carteni, Stefano Mazzoleni, Dong-Hyun Kim

**Affiliations:** Center for Analytical Bioscience, Advanced Materials and Healthcare Technologies Division, School of Pharmacy, University of Nottingham NG7 2RD UK dong-hyun.kim@nottingham.ac.uk; Department of Agricultural Sciences, University of Naples Federico II Portici 80055 Italy

## Abstract

Metabolic flux analysis (MFA) quantitatively describes cellular fluxes to understand metabolic phenotypes and functional behaviour after environmental and/or genetic perturbations. In the last decade, the application of stable isotopes became extremely important to determine and integrate *in vivo* measurements of metabolic reactions in systems biology. ^13^C-MFA is one of the most informative methods used to study central metabolism of biological systems. This review aims to outline the current experimental procedure adopted in ^13^C-MFA, starting from the preparation of cell cultures and labelled tracers to the quenching and extraction of metabolites and their subsequent analysis performed with very powerful software. Here, the limitations and advantages of nuclear magnetic resonance spectroscopy and mass spectrometry techniques used in carbon labelled experiments are elucidated by reviewing the most recent published papers. Furthermore, we summarise the most successful approaches used for computational modelling in flux analysis and the main application areas with a particular focus in metabolic engineering.

## Abstract 2

Metabolic flux analysis (MFA) quantitatively describes cellular fluxes to understand metabolic phenotypes and functional behaviour after environmental and/or genetic perturbations.

## Graphical abstract

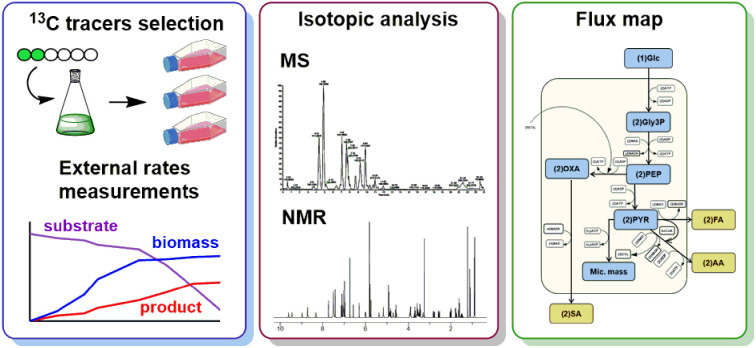

## 1 Introduction

The term “metabolic flux” was used for the first time in the forties by Greenstein and Chalkley,^1^ but only in the 21st century have researchers started using the term “fluxomics”, when all critical parameters of metabolic pathways and enzymatic reactions have been integrated in the analysis of metabolic flux.^2^ Metabolic flux analysis (MFA) is a simultaneous identification and quantification of metabolic fluxes interpreted numerically as the relative fraction of a specific metabolite. Fluxes allow investigation of the effect of genetic and environmental conditions/modifications on a huge set of reactions (including conversion, anabolic, catabolic, uptake and transport of intra and extracellular metabolites) that define the metabolism and physiology of cells.

Several flux analysis techniques have been developed and implemented with very powerful software (*e.g.*, METRAN, INCA, OpenFLUX) for both data collection and data analysis to determine and predict metabolic fluxes with much more accuracy and precision. The selection of specific techniques largely depends on what kind of analysis needs to be performed. Overall, there are seven methods to perform flux analysis: flux balance analysis (FBA), MFA, ^13^C-MFA, isotopic nonstationary MFA (INST-MFA), dynamic MFA (DMFA), ^13^C-DMFA, and COMPLETE-MFA widely discussed in a recent mini-review published in 2015.^3^ Briefly, FBA is the oldest mathematical approach that uses a large-scale model (up to thousand reactions relative to the entire metabolism) and assumes the stationary state of the metabolic network.^4^ This predictive *in silico* model was then simplified in MFA, an easier and smaller-scale model that, in contrast to FBA, is exclusively focused on the central carbon metabolism such as glycolysis, pentose phosphate pathway (PPP), tricarboxylic acid (TCA) cycle,^5^ anaplerotic pathways and gluconeogenesis.^6^ MFA can be now supported by the use of stable isotopes detectable as tracers by mass spectrometry (MS) and nuclear magnetic resonance (NMR) spectroscopy.^7^ In ^13^C-MFA one ^13^C-labelled (*e.g.* [1,2-^13^C] glucose; [1,6-^13^C] glucose; uniformly labelled [U-^13^C] glucose; ^13^C-CO_2_; ^13^C-NaHCO_3_, *etc.*) or multiple singly labelled substrates (COMPLETE-MFA)^8^ are used as the carbon source for cell growth. The ^13^C of the labelled substrate in the medium will be then incorporated into the metabolic network of an organism of interest. This technique aims to identify and quantify flux changes and isotope distribution with the assumption of both metabolic steady state (when all metabolic fluxes remain constant over time) and isotopic steady state (when isotopes are fully incorporated and static). However, one of the limitations of this method is that certain cells (mammalian cells) reach the isotopic steady state relatively late (4 hours or even a day).^9^ To address this issue, ^13^C-INST-MFA allows transient ^13^C-labelling data at the metabolic steady state. In other words, the accumulation of tracers in intracellular metabolites is monitored over time before the system reaches the isotopic steady state but with the assumption of the metabolic steady state.^10^ From an experimental point of view, this method is faster than ^13^C-MFA because it does not need to wait the isotopic stationary state but from a computational point of view, it is much more complex because for each time point it requires solution of differential equations rather than algebraic balance equations. However, using the elementary metabolite unit (EMU) modelling approach is possible to dramatically reduce the computational difficulty.^11,12^ Other options are based on dynamic fluxomics experiments which are highly demanding but provide much more comprehensive information especially if ^13^C-DMFA is applied. The aim of DMFA is to determine changes in fluxes during a culture that is not at the metabolic steady state, thus the experiment is divided into time intervals and for each time interval it is assumed that flux transients are relatively slow. Carbon labelling measurements in dynamic system (^13^C-DMFA) is performed by extrapolating data similarly to ^13^C-MFA technique but splitting the experiment in multiple time points in order to have information of flux transients, which cannot be observed using classical MFA.^13,14^ One of the major limitations of DMFA is the huge amount of data and the complexity of computational models since this technique calculates fluxes for each time interval (from *t*_1_ to *t*_*n*_) and it is based on the assumption that flux transients are relatively slow (on the order of hours instead of seconds or minutes).

To date, among all techniques briefly introduced above, the isotopic stationary ^13^C-MFA is the most applicable and advanced method in biotechnology and systems biology. Because of its relative simplicity, this technique is becoming very popular in many areas, such as medicine, metabolic engineering, biochemistry, biotechnology, and it is the primary approach (a) to determine new metabolic pathways, (b) to predict toxic effects of new drugs, (c) to identify targets after genetic modifications, (d) to explain mechanism of diseases and (e) to optimise biotechnological processes in metabolic engineering.^15–19^ Therefore, since this is an emerging area, we will report the recent advancements in the methods of ^13^C-MFA, various tools available for MFA analysis and its application areas through this systematic review. Mainly we will describe the steps to perform ^13^C-MFA: (a) pre-culture of cells until metabolic steady state and replacement of the medium with a labelled substrate; (b) cell cultivation until isotopic steady state in which molecules incorporate isotopes; (c) extraction of intra and extracellular metabolites for identification and quantification with respect to labelling state using targeted MS or NMR spectroscopy; (d) data processing and computational modelling to evaluate and predict cell fluxes, respectively.

## 2 Results and discussion

**Fig. 1:**
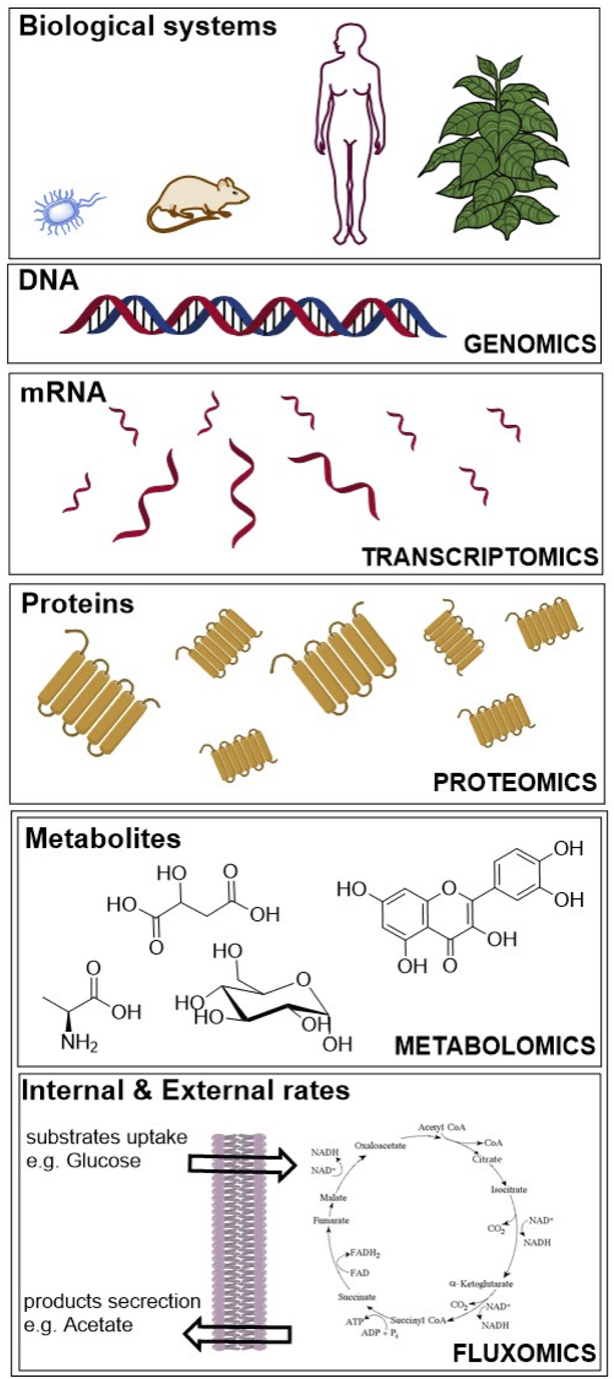
“Omics” fields include genomics, transcriptomics, proteomics, metabolomics and fluxomics, used to investigate biological systems.

We searched journal articles published from 1935 to 2021, concerning the use of fluxomics approach to study biological systems. We used online versions of PubMed, Web of Science and Scopus using the key words, “metabolic flux analysis” OR fluxomics OR ^13^C-MFA. Publications were filtered for English language, duplicates, full text availability and document type (excluding short communications, letters, patents, and book chapters). We used the same electronic databases to survey data analysis and application areas sections. Based on the results obtained we drafted a typical workflow for ^13^C-MFA starting from sample preparation (including labelled solutions and cell cultures), sample treatment for different analytical techniques to data analysis and ending with computational modelling to understand and predict metabolic fluxes. The tools that describe the genotype of biological systems are genomics, transcriptomics and proteomics, which produce data for gene sequence, mRNA and protein abundance, respectively (Fig. 1).^20^

Whereas metabolomics and fluxomics describe the phenotype of cells or organisms providing snapshots of a set of metabolites and metabolic fluxes, respectively, at a given time and conditions. In this review, we focus on the last level of the phenotype description using ^13^C-MFA. Over the past years, different flux techniques have been applied to a wide range of research areas (Table 1).^21–23^

**Table 1:** Different techniques applied in flux analysis^a^

Flux methods	Abbreviation	Labelled tracers	Metabolic steady state	Isotopic steady state
Flux balance analysis	FBA		X	
Metabolic flux analysis	MFA		X	
^13^C-Metabolic flux analysis	^13^C-MFA	X	X	X
Isotopic non-stationary ^13^C-metabolic flux analysis	^13^C-INST-MFA	X	X	
Dynamic metabolic flux analysis	DMFA			
^13^C-Dynamic metabolic flux analysis	^13^C-DMFA	X		
COMPLETA-MFA	COMPLETA-MFA	X	X	X

a Metabolic steady state = metabolic fluxes are assumed to be constant in time, isotopic steady state = isotopes incorporation is assumed to be constant in time.

**Fig. 2:**
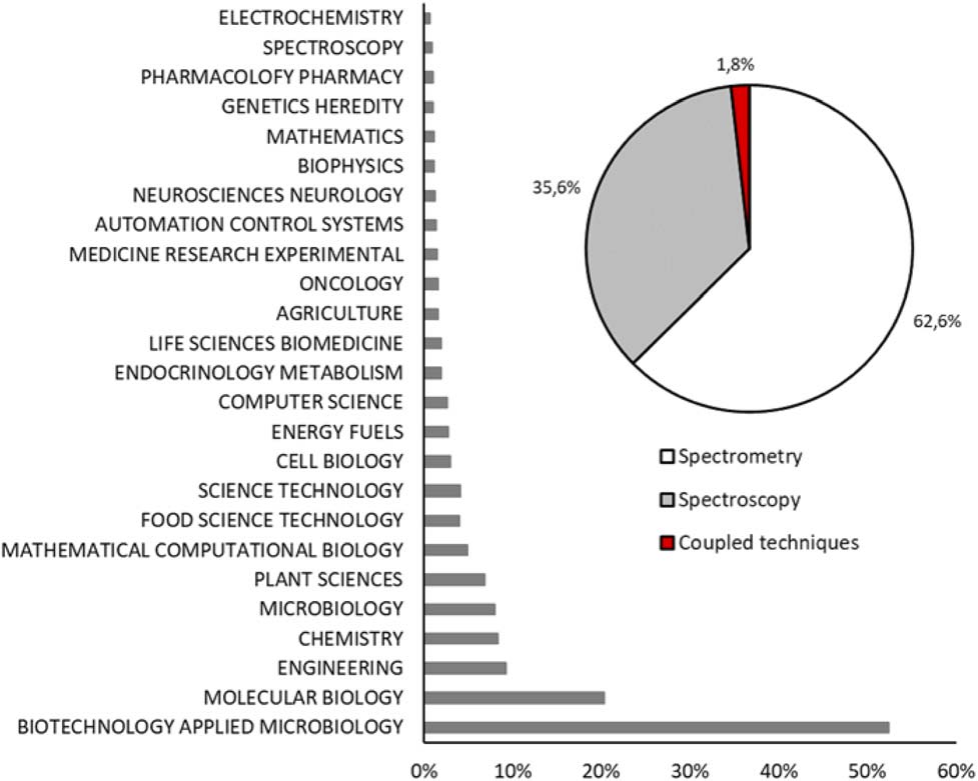
Record count in percentage of articles obtained using key words “metabolic flux analysis OR fluxomics OR ^13^C-MFA” and grouped by main field (histogram) and analytical techniques (pie chart).

Fig. 2 shows the record count in percentage of the scientific papers about MFA grouped in twenty-five research areas. Biotechnology applied to microbiology and molecular biology are the main research areas of fluxomics (52.3% and 20.3%, respectively) followed by engineering (9.2%), chemistry (8.3%) and microbiology (8.0%). According to the reported data, MS is the most used technique to perform flux analysis: 62.6% of scientific papers about MFA are based on MS, while NMR spectroscopy appears in 35.6% of research and 1.8% is represented by those papers in which different techniques are coupled to have a complementary data set.

### 2.1 Sample preparation

The use of stable isotope to study metabolic processes date back to 1935, when Schoenheimer and Rittenberg synthesised deuterated fatty acids to analyse lipid metabolism in animals.^24,25^ Today the most common stable isotopes used in fluxomics are ^2^H, ^13^C, ^15^N and ^18^O (0.015%, 1.11%, 0.365%, and 0.204% relative abundance, respectively). Among them, ^13^C has been mostly used because of its universal presence in each bioorganic molecule and its relative high abundance compared to the corresponding element ^12^C. Deuterium (^2^H or D) has very low natural abundance and it is easily interchangeable as well as ^18^O whereas ^15^N is mostly used to follow the pathway of nitrogen in proteins and nucleic acids. Therefore, because of above mentioned limitations of other isotopes, in this review we focused on the stable incorporation of ^13^C in one or more substrates. One of the first flux measurements using ^13^C NMR was done by Malloy *et al.* in the 1988 who investigated the citric acid cycle of the rat heart.^26^ However, ^13^C-MFA was established in the mid-90s when Zupke and Stephanopoulos presented the first ^13^C flux analysis based on a general mathematical modelling approach.^27^ Then in the 21^st^ century the ^13^C-MFA has widespread also thanks to the development of easy-to-use software such as 13CFLUX.^28^ Sample preparation is based on two main steps; the first one is the preparation of carbon labelling solutions and the second is the feeding of cell cultures using tracers followed by quenching and extraction of metabolites.

#### 2.1.1 Carbon labelling experiments

**Fig. 3:**
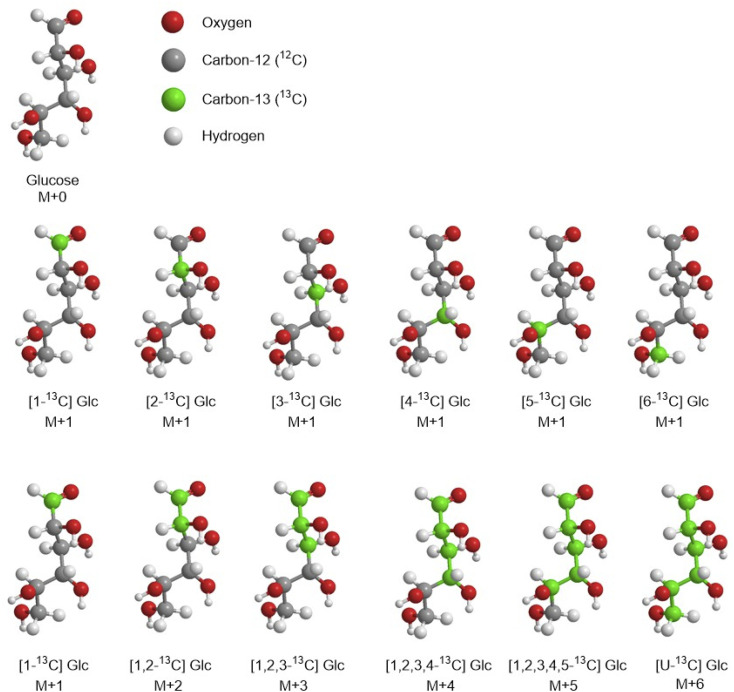
Example of some isotopomers and isotopologues of the glucose.

In carbon labelling experiments (CLE) one or more substrates are used to feed biological systems. The tracers are used as carbon source for cell growth and isotopes are immediately incorporated in cell metabolism. Because each carbon of the molecule can be labelled at each position, for the same molecule, there are multiple carbon labelling substrates called isotopomers. For example, considering ^12^C, one of its isotopes is ^13^C which can be incorporated in glucose (Glc) in six different positions giving 6 different isotopomers singly labelled and called [1-^13^C], [2-^13^C], [3-^13^C], [4-^13^C], [5-^13^C] and [6-^13^C] Glc (Fig. 3). Additionally, it is also possible to have more than one ^13^C isotopes in the same molecule. As an example, in [1,2-^13^C] Glc the ^12^C in position C1 and C2 is replaced by ^13^C. In mass spectrometry this is indicated as M+2 or M_2_ meaning that [1,2-^13^C] Glc differs from the unlabelled glucose of 2 mass units. The M+0 (unlabelled molecule), M+1, M+2, M+3, *etc.* are different isotopologues of the same molecule. In other words, the isotopomers differ only in the position of an isotope, while the isotopologues differ in the isotopic composition. The numbers of isotopomers and isotopologues dramatically increase when hydrogen and oxygen isotopes are included in the molecule. It is very important to understand that in both unlabelled substrates and labelled substrates there is 1.11% of probability to find the ^13^C, because of its natural abundance, percentage to take in consideration as correction factor in ^13^C-MFA experiments. This is why the unlabelled molecules are also identified as “naturally labelled”.

Isotopologues can be easily obtained by commercial suppliers, but they are very expensive *e.g.*, for 1 g of [1,2-^13^C] glucose the price can range between £500 and £800, 1 g of [5,6-^13^C] glucose is commercially available for £2300 (https://www.omicronbio.com/index.html). The most common glucose isotopomers used for CLE are [1-^13^C] glucose and [U-^13^C] glucose (uniformly labelled). However, to obtain a comprehensive and accurate flux map of a biological system the selection of appropriate ^13^C-tracers is an important step in ^13^C-MFA. There are four ways to select the best tracer in CLE, the first option is based on a comprehensive literature research. The investigator extrapolates and compares data on different labelled substrates used for studying the same metabolic pathway of the same organism (or similar) to select those one that achieved the best precisions. This option based on data obtained from different laboratories is the cheapest and fastest approach. The second option is based only on empirical experiments which might be a long path and many experiments might be run before finding the optimal tracer and so it can be very expensive considering the price of 1 g of a specific tracer such as [1-^13^C] glucose and [U-^13^C] glucose (∼£300 per g), [2-^13^C] glucose (∼£700 per g) and [1,2-^13^C] glucose (∼£900 per g). The third option is based on practical experiments at first followed by simulation experiments, for example Metallo *et al.* have experimentally determined metabolic fluxes in a tumour cell line and then they have computationally evaluated a wide selection of labelled ^13^C glucose tracers for their ability to precisely estimate fluxes in central carbon metabolism. In the end they identified as optimal tracer [1,2-^13^C] Glc for glycolysis, PPP, and the overall network.^29^ The fourth option is to evaluate *a priori* all possible tracer combinations using simulations and predictive approaches. Hence, Crown and Antoniewicz introduced a new framework for optimal ^13^C-tracer experiment design based on elementary metabolite units (EMU) decomposition of metabolic network models, in which a measured metabolite is decomposed into a linear combination of so called EMU basis vectors (EMU-BV), where each EMU-BV represents a unique way of assembling substrate's EMUs into measured metabolites (the EMU framework is discussed in more detail in the section “Computational modelling and data analysis software”).^30^ With this methodology the same group achieved the identification of two optimal tracers [2,3,4,5,6-^13^C] glucose and [3,4-^13^C] glucose for elucidating the oxidative PPP flux and pyruvate carboxylase flux, respectively in mammalian cells.^31,33^ Later on, in another work they evaluated 100 random flux maps for commercially available glucose tracers on single, mixed, and parallel CLE having *E. coli* as object of the study.^32^ Results showed the best tracers that achieved the highest precision for flux analysis are [1,2-^13^C], [1,6-^13^C] and [5,6-^13^C] glucose.

Many other substrates other than glucose can be used in CLE as carbon source, such as glutamine, carbon dioxide, glycerol, acetate, *etc.* (Table 2). Since metabolic pathways utilise substrates atoms in a specific and predictable way, the choice of the tracer also depends on the studied metabolic pathways and organisms of interest. Yoo *et al.* reported that glutamine is a major source of carbon for fatty acid synthesis in a brown adipocyte cell line. Later, they investigated the pathway for fatty acid synthesis from glutamine using [U-^13^C] glutamine or [5-^13^C] glutamine to quantify fluxes and analysed the mass isotopomer distribution (a number that indicates the relative amount of each mass isotopomer, more details in the paragraph “GC-MS”) in wild type brown preadipocytes.^34^ Metallo *et al.* experimentally determined metabolic fluxes in A549 lung carcinoma cell line using different ^13^C-labelled glucose and glutamine tracers.^35^ Results showed that [1,2-^13^C] glucose provided the most precise estimates for glycolysis and PPP, while [U-^13^C] glutamine emerged as the preferred isotopic tracer for the analysis of TCA cycle. Kempa *et al.* used ^13^CO_2_ and ^13^C-acetate to study the uptake in the unicellular green alga *Chlamydomonas reinhardtii* in both photoautotrophic and mixotrophic growth conditions, respectively.^36^ Very recently, Tomàs-Gamisans *et al.* used 20% [1,3-^13^C] glycerol as sole C-source in the steady state CLE to obtain a more reliable estimation of metabolic fluxes through the central carbon pathways of *Pichia pastoris* culture.^37^

**Table 2:** Metabolic flux methods applied to different organisms. Isotopic tracers used in the experiments, techniques and software for data analysis are reported as well. When authors did not use “omics” software to analyse data the abbreviation “ns” is reported for “not specified”^a^

Flux methods & isotopomers	Organisms	Techniques	Software data analysis	Ref.
^**13**^ **C-MFA**				
80% unlabelled Glc and 20% [U-^13^C] Glc	*S. cerevisiae*, *S. bayanus* var. *uvarum*, *S. exiguus*, *S. servazzii*, *Z. rouxii*, *S. kluyveri K. thermotolerans K. lactis K. marxianus* var. *marxianus*, *P. angusta*, *D. hansenii*, *P. sorbitophila*, *C. tropicalis*, *Y. lipolytica*	GC-MS	METAFoR	38
[1-^13^C] Glc or a mix of 20% [U-^13^C] Glc and 80% unlabelled Glc	*E. coli*	GC-MS	METAFoR	39
5 mM Glc + 5 mM [2-^13^C] acetate, 5 mM [1-^13^C] Glc + 5 U/L insulin and 5 mM Glc + [3-^13^C] pyruvate	Rat hearts	GC-MS along with ^13^C-NMR (400 MHz)	ns	40
0%, 0.5%, 1%, 2% and 10% of [1-^13^C] Glc mixture	*C. glutamicum*	GC-C-IRMS	MATLAB	41
^13^C sodium acetate; ^13^C sodium hydrogen carbonate	*Chlamydomonas reinhardtii*	GCxGC-TOF-MS	MetMax and GMD	36
[1-^13^C] Glc; 99% [6-^13^C] Glc	*C. glutamicum*	MALDI-TOF MS		42
[1,2-^13^C] Glc, [1,6-^13^C] Glc	*E. coli*	GC-MS	METRAN	43
[5-^13^C] glutamine and [U-^13^C] glutamine	Brown adipocyte cells	GC-MS	METRAN and ISA	34
[1-^13^C] Glc	*C. glutamicum*	GC-MS	OpenFLUX	44
[1-^13^C] Glc, or a mix of 20% [U-^13^C] Glc and 80% naturally labelled Glc	*E. coli*, *B. subtilis*	GC-MS	SUMOFLUX, INCA	45
25 mM 1 : 1 mixture of [U-^13^C] Glc and [1-^13^C] Glc	*A549 lung carcinoma cell line*	GC-MS	METRAN	35
2 g L^−1^ NaH^13^CO_3_ and 5 g L^−1^ Glc (U-^13^C_6_ or 1-^13^C_1_)	*Synechocystis* sp. PCC 6803	GC-MS	MATLAB	46
20% [1,3-^13^C]-glycerol and 80% unlabeled glycerol	*Pichia pastoris* X-33 strain	GC-MS	OpenFLUX	37
[1,2-^13^C] Glc	Co-culture of *E. coli* Δ*pgi* and Δ*zwf*	GC-MS	METRAN	47
[l-^13^C] Glc and [U-^13^C] ethanol	*S. cerevisiae* strain CEN.PK-113.7D	LC-MS	MATLAB	48
500 g L^−1^ 1 : 1 mixture of [1-^13^C] Glc and [U-^13^C] Glc	*E. coli* strain WYK050	LC-MS/MS	MATLAB	49
A mixture of 80% [1-^13^C] Glc and 20% [U-^13^C] Glc	*B. subtilis* strain BSB168 *trp*^*+*^	LC-MS/MS	13CFLUX	50
[1-^13^C] Glc and [U-^13^C] Glc	*E. coli* strain K-12 MG1655	LC-MS/MS	MATLAB and INCA	51
[l-^13^C] Glc	*Corynebacterium glutamicum*	^1^H-^13^C NMR	ns	52
[U-^13^C] Glc 10% and natural labeled Glc 90%	*B. subtilis*	^1^H-^13^C NMR	ns	53
13.9 mM of a mixture of either 20% [U-^13^C]-Glc and 80% [1-^13^C]-Glc or [U-^13^C]-Xyl and 80% [1-^13^C]-Xyl	*E. coli* Pzwf1.1, Pzwf1.2, Pzwf1.3, Pzwf1.4 and *E. coli* Pzwf1, Pzwf2, Pzwf3, Pzwf4	^1^H-^13^C NMR	influx_s	54
[1,2-^13^C] Glc	Mammalian cells	^1^H-^13^C NMR coupled with GC-MS	ns	55
90% unlabelled Glc and 10% uniformly labeled [U-^13^C] Glc	*Synechocystis*	^1^H-^13^C NMR coupled with GC-MS	ns	56
[^13^C_2_] glycine, [^13^C_3_] serine and [3-^13^C] serine	*A. gossypii* B2	GC-MS, LC-MS, and NMR	OpenFLUX	57
[U-^13^C] Glc 10%, [1-^13^C] Glc 40% and 50% unlabelled Glc	Chinese Hamster Ovary (CHO) cells	2D [^13^C, ^1^H] COSY	13C-Flux	58
50 mM [1-^13^C] Glc	*Actinobacillus succinogenes*	GC-MS, ^1^H-^13^C NMR	13C-Flux	59
20% [U-^13^C] Glc and 80% unlabelled Glc	*Escherichia coli*	3D TOCSY-HSQC	ns	60

**FBA**				
[1-^13^C] Glc	*Geobacillus thermoglucosidasius* M10EXG	Based on GC-MS data	SimPheny Software from Genomatica	61
	Chinese Hamster Ovary (CHO) cells	Based on 2D [^13^C, ^1^H] COSY data	13C-Flux	58

^**13**^ **C-INST-MFA**				
[1,6-^13^C] Glc	Rat brain (*in vivo*)	^13^C-NMR	ns	62

a Ns = not specified, Glc = glucose; GC-C-IRMS = gas chromatography-combustion-isotope ratio mass spectrometry; GCxGC-TOF-MS = two-dimensional gas chromatography coupled to time-of-flight mass spectrometry; GMD = Golm metabolome database; Glc = glucose; Xyl = xylose.

The solution of labelled tracers is typically prepared in deionised water and then filtered with 0.22 μm filters. It can be stored at 4 °C until use and in the meanwhile the investigator can proceed with cell pre-cultures.

#### 2.1.2 Cell cultures

**Fig. 4:**
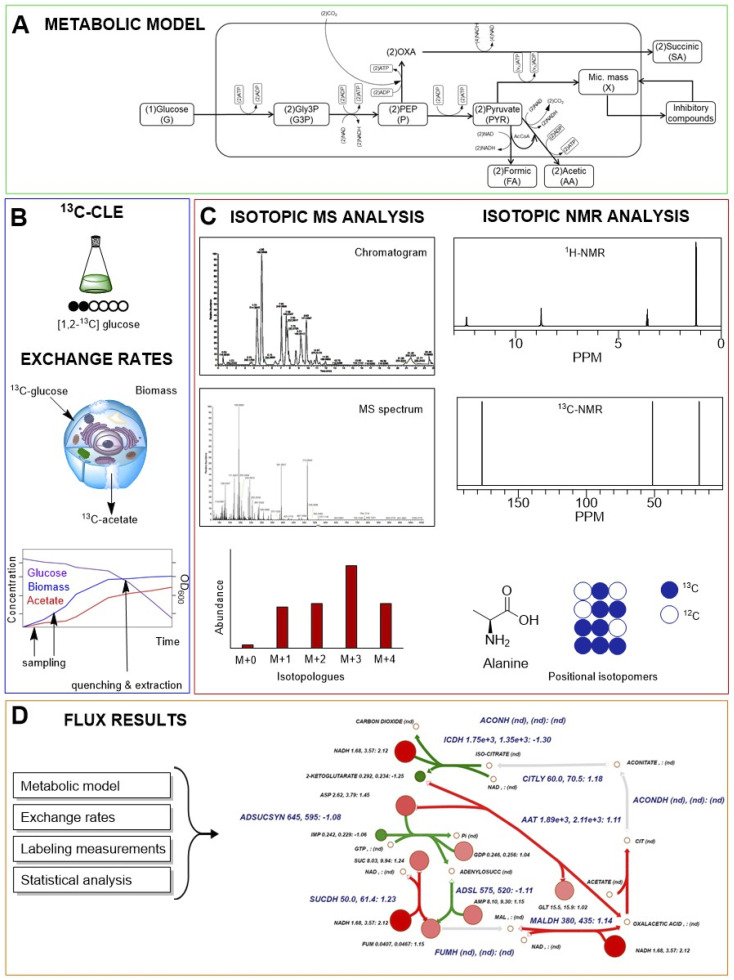
A typical carbon labelling experiment (CLE) workflow. (A) A metabolic model of cellular metabolism is predicted. (B) Cell cultivation and ^13^C-CLE are conducted, sampling is performed to have information about exchange rates between cells and environment; when metabolic steady state and isotopic steady are reached, quenching and extraction are carried out. (C) MS or NMR spectroscopy-based isotopic analysis are performed for labelling measurements. (D) Cell fluxes are assessed by integration of internal and external rate calculations.

Experimental procedure for the cultivation of different cells is well documented in the literature. Researchers all over the world reported data from the well-studied prokaryotic model organism *E. coli* to more complex systems such as human cells and plants.^63,64^ Whatever is the type of cells chosen for the experiments after the preparation of labelled solution, the second step is the pre-culture of cells in unlabelled medium. For example, regarding microorganisms, continuous cultures in bioreactor (typically chemostat or turbidostat between 30 and 1000 mL) are the preferred method to achieve maximum reproducibility and to maintain the metabolic steady state during the isotope incorporation. Isotope tracer solution is introduced by switching the medium from unlabelled to labelled at the same concentration to minimise cell's perturbation and maintain the stationary state. Alternatively, the exponential growth phase in batch cultures mimics the metabolic steady state. In this case, the procedure requires the inoculum of the species (typically at a low cell density OD_600_ ∼ 0.01) from unlabelled pre-culture to labelled medium when the optical density measurements indicate the stationary metabolic state (Fig. 4). As mentioned in the previous section, the modified medium should not contain any source of ^12^C. This is easy to achieve when the selected species use only organic molecules as a carbon source, such as glucose and glutamine, but for microorganisms that can fix ^12^CO_2_ from the atmosphere, such as cyanobacteria and algae, it is more difficult to avoid ^12^C contamination that can lead to alteration of ^13^C-MFA results because of ^12^C incorporation in the cell metabolism. You *et al.* delineated the photomixotrophic metabolism of *Synechocystis* sp. PCC 6803 grown in modified BG-11 medium in which ^13^C was supplied by 2 g L^−1^ NaH^13^CO_3_ and 5 g L^−1^ glucose (U-^13^C_6_ or 1-^13^C_1_) and ferric ammonium citrate was replaced by ferric ammonium sulfate.^46^ The serum bottles were sealed with rubber septa to prevent atmospheric CO_2_ intrusion immediately after the inoculum of unlabelled *Synechocystis* 6803 (at OD_730_ = 0.9) in 30 mL ^13^C-labelled medium.

During isotope incorporation, the ^13^C tracer is quickly distributed all over the metabolic pathways until the isotopic steady state is reached in all cells. In this phase, the growth rate, uptake rate, and biomass must be quantified (Fig. 4).^43^

The final samples are collected when metabolic and isotopic steady state are reached. The collection of samples is a critical step because all metabolic reactions need to be stopped as quickly as possible and any leak of metabolites must be avoided during the extraction. Ideally, the selected method should extract the higher number of metabolites without compromise their chemical or physical properties. Quenching and extraction methods can slightly change, depending on cell types and metabolites of interest.^65–68^ The first methods for quenching and extraction date back to 70s and were based on the use of hot water and boiling ethanol.^69^ Then in the early 90s, to reduce the degradation of thermolabile compounds such as, phosphorylates and nucleotides, the addition of cold solvent (−20 °C or below such as −40 °C or −70 °C) on cell cultures, typically methanol, became the most common applied quenching method.^5,70^ Alternatively, to decrease the risk of cell lysis and the subsequent leak of intracellular metabolites in the medium, rapid quenching of cells by immersion of culture flasks in a dry ice–ethanol bath at 0 °C, has been proposed by Robinson *et al.*^71^ The application of acidic or alkaline conditions to extract metabolites has also been proposed in the 90s.^72^ Later on, Maharjan and Ferenci compared the metabolome of *E. coli* extracted with six different methods: hot ethanol (90 °C), hot methanol (70 °C), cold methanol (−20 °C), perchloric acid, potassium hydroxide, and methanol/chloroform.^73^ Results showed that higher temperature methods give loss of compounds because of thermal degradation while the use of extreme pH is more suitable for extraction of nucleotides, but compounds like pyruvate, NAD, NADH^+^ are unstable at these conditions. Thus, they selected the cold methanol approach as the best method for metabolome extraction. More recently, Prasannan *et al.* presented a new method for metabolites extraction from three strains of cyanobacteria (*Synechococcus* sp. PCC 7002, *Synechococcus elongatus* PCC 7942, and PCC 11801).^74^ To reduce the risk of metabolite leak in the medium during quenching, the improved method is based on fast filtration to separate cells from supernatant, subsequent immersion of the filter in cold methanol and the use of a water–methanol–chloroform–NH_4_OH system for metabolite extraction. Data showed that the addition of NH_4_OH to water/methanol/chloroform solution increased the intensity of metabolite classes such as sugar phosphates, bisphosphates, and nucleotide triphosphates.^74^ Although, the use of weak base did not improve the extraction of organic acids, they observed an increase of fatty acids and amino acids into the aqueous layer. In ^13^C-MFA both isotopic labelling and external rates, such as substrate uptake rates, product secretion rates and growth rates, must be quantified to obtain essential constraints for flux determination. Therefore, the separation of the spent growth medium from cell pellet is a necessary step in ^13^C-MFA to analyse intra and extra-cellular metabolites. There are two main cell collection methods: fast filtration, as previously mentioned, and centrifugation at high speed (*e.g.*, 14 000 g) typically performed after quenching. Analysis of external rates and product yields (such as acetate, lactate, propionate, ethanol or other by-products) might be challenging due to low concentrations of extracellular metabolites. For an excellent discussion on how to determine external rate and yields see Box 2 of the paper in Nature Protocols by Long and Antoniewicz.^43^ Regarding the intracellular metabolites, researchers focused their attention on central metabolic intermediates and mostly on proteinogenic amino acids since they reflect the labelling patterns of multiple key metabolites at steady state. To extract intracellular metabolites cell hydrolysis is required and this mostly depends on the type of cells and the biological system studied. Typically, to perform cell hydrolysis the pellet is mixed with a strong acid at high temperature, for example it has been reported for *Corynebacterium glutamicum* and *E. coli* that 0.5 mL of 6 M HCl and incubation for 24 h at 110 °C is a good method for intracellular metabolites extraction.^41,75^ For cyanobacteria different solvent systems used for extraction led to statistically significant changes in the extraction efficiency for a large number of metabolites.^74^ Thus, cell lysis depends on the membrane structure which can differ from bacteria (Gram negative and Gram positive), yeasts, plants, to eukaryotic and prokaryotic cells. In each case, to minimise degradation after extraction, samples can be either dried under vacuum and stored in a desiccator at room temperature for up to 4 days or directly stored at −80 °C (for a year) until further treatments.^43^ An extremely rapid sampling technique for high throughput fluxome profiling has been developed by Heux *et al.*^54^ They presented an automated cultivation and sampling platform for CLE composed by a bioreactor and an automatic workstation.^76,77^ The bioreactor has a capacity of 48 stirred tank (from 8 to 15 mL) equipped with solid state sensors for oxygen and pH. The robot workstation, activated *via* software, provides sterile conditions and is composed by three robotic arms to handle liquids, transport tubes to/from centrifuge and filter station, microtiter plates, a plat reader, a barcode reader, a cooling and heating module. This platform has been applied to discriminate a set of *E. coli* mutants with varying level of glucose-6-phosphate dehydrogenase by NMR-based isotopic profiling. With this automated system, they generated 80 fluxes of *E. coli* mutants (10 strains) in only 4 days, including all steps from cultivation to calculated fluxes. It is important to mention that the work published by Shaikh *et al.* in 2008 provided the foundation to extend isotopomer-based flux analysis to study metabolism of individual strains in microbial communities.^78^ Then in 2011 Rühl *et al.* developed a method for ^13^C flux analysis of subpopulations within mixed cultures of *E. coli* (wild type with two metabolic mutants) using a plasmid-based reporter protein with glutathione *S*-transferase (GST) as the high-affinity purification tag and green fluorescent protein (GFP).^79^ These methods required physical separation of either proteins or cells, that could lead to inaccurate flux results. To overcome this issue, a novel approach for performing ^13^C-MFA of co-culture systems was presented by Gebreselassie and Antoniewicz in 2015.^47^ This approach did not require any physical separation to measure species-specific fluxes, but a careful selection of isotopic tracers was needed. The methodology was validated using as tracer [1,2-^13^C] Glc and a co-culture of two *E. coli* knockout strains Δ*pgi* (knockout of upper glycolysis) and Δ*zwf* (knockout of oxidative PPP).^47^ This method was lately employed to investigate for the first time the metabolism of *E. coli* grown on agar that formed two distinct cell populations engaged in acetate cross-feeding.^80^ Given the great importance of the synergistic functions of microbial communities, these works have extended the scope of ^13^C-MFA to a large number of multi-cellular systems.

### 2.2 Sample treatment and analytical techniques

Multiple analytical techniques can be applied in MFA. After the extraction of metabolites, samples are treated differently based on which analytical technique has been selected for flux analysis. The most common used techniques are MS and NMR spectroscopy. For each technique, there are advantages and disadvantages to take in consideration before starting the analysis. In general, it is difficult to apply NMR to extracellular metabolites because of their relatively low amount, while MS provides more information because of its higher sensitivity.^81^ The range for the sensitivity depends on the type of instruments, usually accurate NMR measurements are possible in the concentration range 10^−1^ to 10^−3^ M, with structural elucidation still being possible at concentrations of approximately 10^−4^ to 10^−5^ M. Electron impact (EI)-MS is more sensitive and can operate at concentrations of 10^−6^ M, while commonly available electrospray ionisation (ESI)-MS can be efficient up to a concentration of 10^−12^ M.^82^ The possibility to choose between a full scan, selective ion monitoring, single reaction monitoring (SRM), multiple reaction monitoring (MRM) and MS/MS fragmentation allow to obtain more information about isotope patterns. In addition, with these techniques, the molecular formula can be directly determined without previous purification because of the high mass resolution of the instrument (*e.g.*, Fourier transform-ion cyclotron resonance-MS or Orbitrap instrument). On the other hand, the major advantage of NMR is that this technique is not destructive; samples can be used again after NMR analysis. NMR spectroscopy and MS are often complementary to each other and provide different information, for example, NMR spectroscopy can provide detailed isotopic labelling position for each specific carbon in a metabolite of interest and MS can provide the mass isotopomer distribution (MID). Therefore, the combined approach is highly suggested to obtain more precision and accuracy of results.^55,56^

#### 2.2.1 NMR spectroscopy

Nuclear magnetic resonance spectroscopy is a non-destructive analytical technique and one of the first method applied in CLE.^52^ Within NMR analytical techniques, proton and carbon NMR are the most common one (Table 2). Mono-dimensional experiments can be sometimes very laborious to resolve especially for crowded region of the spectra where many overlapped peaks make metabolites identification difficult. To overcome this issue, two-dimensional NMR analysis with both homonuclear and heteronuclear, such as ^1^H-^1^H COSY, ^1^H-^1^H NOESY, ^1^H-^13^C HSQC, ^1^H-^13^C HMBC, have been applied in ^13^C-MFA to analyse several metabolites in a complex mixture without purification.^83^ Sometimes, even in two-dimensional NMR spectra the chemical shift of different molecules can be overlapped by other diagnostic peaks, and the acquisition of 3D spectrum requires very long time, from 3 to 7 days. To reduce the acquisition time of analysis to a few hours, Reardon *et al.* proposed the use of a 3D TOCSY-HSQC experiment based on Non-Uniform Sampling (NUS), making 3D experiments suitable for ^13^C-MFA.^60^ Data showed that this new strategy is linear and quantitative. The authors were able to provide valuable site-specific ^13^C enrichment information. This is one of the major advantages of NMR as specific positional information for each carbon atom can be obtained without chemical degradation of metabolites allowing to quantify the abundance of positional isotopomers. Moreover, because of its non-invasive approach, NMR can be also used to evaluate metabolism *in vivo* study. Des Rosiers *et al.* determined cardiac metabolism in *ex vivo* rat heart perfusion with ^13^C-substrates.^84^ A more recent *in vivo* study was reported by Jeffrey *et al.* who measured metabolic fluxes in the rat brain using the additional information provided by ^13^C multiplets.^62^ However, for NMR analysis metabolites must be at least in μM quantities, thus a relatively large amount of sample is needed compared to MS analysis.

Sample treatment for NMR analysis does not require long time as for GC-MS analysis because there is no need of sample derivatisation for spectroscopy. On the other hand, NMR analysis can take much longer than MS analysis (several hours of acquisition for one carbon experiment if metabolites are present at the very low concentrations). Once the sample has been dried (see “cell cultures” section), it is dissolved in a deuterated solvent with buffering agent (*e.g.*, potassium dihydrogen phosphate, sodium phosphate). A typical sample for proton spectroscopy in 5 mm NMR tube ranges between 500–700 μL and it is composed by 0.02% sodium azide in ^2^H_2_O with 0.5 mM internal standard like 4,4-dimethyl-4-silapentane-1-sulfonic acid.^85–87^ Sodium azide limits the growth of microorganism in samples if longer experiments are needed, phosphate buffer helps to control the pH of solutions to reduce possible shifts in the spectra between samples and DSS is used as reference standard to set its singlet at *δ* = 0.00 for proton experiments. A centrifuge step is required to remove salt precipitations in the tube. Experiments are normally acquired at 40 °C on 400, 500, 600 and 700 MHz spectrometer depending on the availability of laboratories.^53,88^ Then, after data acquisition, sample can be dried again and used for further analysis, for example MS-based approach.

Bacher *et al.* illustrated a method to obtain metabolite flux patterns of central metabolic pools (carbohydrate phosphates, dicarboxylic acids and acetyl CoA) by biosynthetic retro analysis using NMR spectroscopy.^89^ They used the mevalonate pathway and the deoxy xylulose pathway of terpenoid biosynthesis as examples.

Schwechheimer *et al.* studied the fungus *Ashbya gossypii* to improve its production of riboflavin (vitamin B2), a very important precursor for the cofactors FAD and FMN, from vegetable oil as raw material.^57^ They used a highly sophisticated MFA approach based on parallel CLE (with glycine and serine as tracers) using GC-MS, LC-MS, 1D, and 2D NMR to resolve carbon fluxes. Results explained a complex industrial process showing that glycine was exclusively used as carbon-two donor of the vitamin's pyrimidine ring, while formate and serine do not play an important role in riboflavin biosynthesis.

Goudar *et al.* compared fluxes estimates from the metabolite balancing and isotope tracer methods for Chinese hamster ovary (CHO) cells using ^13^C glucose and 2D-NMR spectroscopy.^58^ They observed good agreement between the 2 methods in the glycolytic, TCA cycle and oxidative phosphorylation fluxes with less than 8% difference for most fluxes (except for PPP and anaplerotic conversion of pyruvate to oxaloacetate fluxes which can only be estimated by the isotope tracer method). This data suggests that relatively little labelling information is needed to get a complete picture of metabolic network. Thus, their study can be used for routine monitoring and for bioprocess development experiments without the need for frequent isotope labelling experiments reducing the analytical investment.

McKinlay *et al.* determined metabolic fluxes from he *Actinobacillus succinogenes* (a promising candidate for industrial succinate production) by using a combined approach based on GC-MS and NMR spectroscopy to understand carbon flux distribution to succinate and alternative products.^59^ They described the labelled experiment based on [1-^13^C] glucose to obtain metabolic pathways of *A. succinogenes* by analyses of its cell composition, extracellular fluxes, and isotopomers of amino acids, organic acids, and glycogen monomers. In particular, for NMR analysis all compounds were analysed by ^1^H-NMR and ^1^H decoupled ^13^C-NMR using a Varian VXR 500 MHz spectrometer. They found that the partitioning of flux between succinate and alternative fermentation products can occur at multiple nodes in *A. succinogenes* and metabolic engineering strategies can be easily designed to increase succinate production.

#### 2.2.2 GC-MS

Mass spectrometry-based methods are a great alternative to spectroscopy techniques, ensuring efficiency, precisions, accuracy, and high sensitivity (Table 2). However, sample treatment for GC-MS can be more demanding than NMR due to derivatisation procedures, which allow metabolites to be more volatile and stable at high temperature of gas chromatography. Derivatisation procedures consist in a simple reaction between dried sample and derivatising agent at precise time and temperature to achieve silylation, acylation, esterification, alkylation or methoxymation. Caution should be paid in this step; in fact, it is very important to start the derivatisation reaction with samples completely dried, since the presence of water will cause hydrolysis of derivatised products with subsequent formation of initial reagents (less volatile and less stable at GC-MS). The positive aspect of the derivatisation step is the lower background noise and thus higher signal to noise ratios in GC-MS spectra compared to those one obtained from LC-MS. This is one of the main reasons why GC-MS technique is the preferred choice for MFA.

Thus, for proteinogenic amino acids analysis, after cell hydrolysis, the lyophilised sample needs to be further solubilised in a derivatising agent such as *N*-methyl-*N-t*-butyl dimethyl silyl-trifluoroacetamide (MBDSTFA) or *N-tert*-butyl dimethyl silyl-*N*-methyl trifluoro acetamide with 1% (wt/wt) *tert*-butyl dimethyl chloro silane (MTBSTFA + 1% TBDMSClS) and leave 30 min at 60 °C for reaction to be completed. Time and temperature should be monitored over time to verify the completion reaction, for example sample injection after 30 min and 60 min at the same temperature (60 °C) should be compared between them and another high temperature (*e.g.*, 80 °C) should be verified to choose the best solution for the samples. Table 3 summarises the main GC-MS-based ^13^C-MFA approaches, including different derivatisation methods.

**Table 3:** GC-MS approaches reported for ^13^C-MFA with relative derivatization methods and instrument characteristics for different target compounds^a^

Instrument	Column	Oven temperature	Derivatizing agent	Target compounds	Ref.
Thermo-Quest ion trap GC-MS, mass tandem (70 eV)	DB5 ms (30 m × 0.25 mm × 0.25 μm)	*T* = 150 °C; increased by 3 °C min^−1^ to 180 °C, followed by a ramp of 40 °C min^−1^ to 260 °C	Dimethyl formamide dimethyl acetal (methyl 8)	Glutamate, citric acid cycle metabolites	40
HP6890 GC with GC/C III interface with a Ni/Cu/Pt combustion reactor and MAT 253 gas isotope MS (77 eV)	DB1 (60 m × 0.25 mm × 0.1 μm)	*T* = 120 °C for 5 min; increased by 5 °C min^−1^ to 280 °C, followed by a ramp of 20°C min^−1^ to 310 °C and kept isotherm for 5 min	100 μL of MBDSTFA (80 °C for 60 min)	Proteinogenic amino acid	41 and 75
Agilent GC 7890B connected to a MS single quadrupole 5977A (EI-70 eV)	DB-5 ms (30 m × 0.25 mm × 0.25 μm)	*T* = 80 °C for 2 min, increased by 7 °C min^−1^ to 280 °C, hold for 20 min	35 μL of pyridine and 50 μL of MTBSTFA + 1% (wt/wt) TBDMSClS (60 °C for 30 min)	Amino acids	43
			50 μL of 2% (wt/vol) hydroxylamine hydrochloride in pyridine (90 °C for 1 h). Then add 100 μL of propionic anhydride (60 °C for 30 min)	Carbohydrates	
			1 mL MeOH and 50 μL of concentrated sulfuric acid (100 °C for 2 h). Add 1.5 mL of DI water and 3 mL of hexane. Centrifuge, separate phases, evaporate to dryness and dissolve in 100 μL of hexane	FAMEs	
GC connected to an HP5971 MSD operating under ionization by electron impact at 70 eV	DB-XLB (60 m × 0.25 mm × 0.25 μm)	*T* = 100 °C for 5 min, increased by 10 °C min^−1^ to 300 °C and hold for 5 min	70 μL of MTBSTFA (70 °C f or 30 min)	Organic and amino acids	34
GC 6890 connected to MS 5973	DB5 column	ns	100 μL THF and 100 μL MTBSTFA (70 °C for 1 h)	Amino acids	61
GC 7890B connected to MS 5875A, Agilent	HP-5-MS (30 m × 0.25 mm × 0.25 μm)	ns	0.1% pyridine in MBDSTFA	Amino acids	57
Agilent 5973 inert MSD benchtop quadrupole mass spectrometer	DB5 ms column (60 m × 0.25 mm × 0.25 μm)	*T* = 100 °C for 4 min, increased by 5 °C min^−1^ to 200 °C, then by 10 °C min^−1^ to 300 °C, and held at 300 °C for 10 min	MTBSTFA at 25 °C for 30 min, then at 120 °C for 1 h	Amino acids and organic acids	59
Agilent 6890 GC connected to Agilent 5975B MS (EI 70 eV)	DB-35 MS capillary column	*T* = 100 °C for 3 min, increased by 3.5 °C min^−1^ to 300 °C	60 μL of 2% methoxyamine hydrochloride in pyridine at 37 °C for 2 h, then 90 μL MBTSTFA + 1% TBDMCS at 55 °C for 60 min	Central carbon metabolism	35
Agilent 6890 gas chromatograph coupled to an Agilent 5973 quadruple MS	Equity®-1701 (15 m, 0.25 mm i.d., 0.25 μm film)	*T* = 75 °C for 1 min. increased by 40 °C min^−1^ to 165 °C, then by 4 °C min^−1^ to 190 °C and then 40 °C min^−1^ to 240 °C. At the end, temperature was increased by 4 °C min^−1^ to 260 °C and held constant for 4 min	55 μL ECF	Proteinogenic amino acid	37

a Central metabolism = glycolysis, tricarboxylic acid cycle (TCA) or citric acid cycle (CAC) or Krebs cycle, pentose phosphate pathway (PPP); THF = tetrahydrofuran; MTBSTFA = *N*-(tert-butyldimethylsilyl)-*N*-methyltrifluoroacetamide; FAME = fatty acids methyl ester; *t*-BDMS = *t*-butyldimethylsilyl; ECF = *N*-ethoxycarbonyl-amino ethyl-esters.

For analysis of sugars, since the silylation of sugars usually results in five tautomeric forms (one open chain, α and β pyranose, and α and β furanose) of the reducing sugars, before silylation another reaction called oxymation is required. The high number of tautomers that silylation would form, would cause major problems for the identification and quantification of complex mixtures as in the case of MFA. However, by converting the aldehyde and keto groups of saccharides into oximes, the number of tautomeric forms can be reduced, due to the C=N bond, resulting only in the formation of *syn* and *anti* forms.^90^ Also the oxymation reaction needs to be performed with dried sample and it is generally carried out by adding 2% (wt/vol) hydroxylamine hydrochloride or methoxyamine hydrochloride in anhydrous pyridine to lyophilised sample and leaving for 1 h at 90 °C (also in this case time and temperature should be verified, Table 3). The oxymated product needs to be further derivatised with MSTFA or propionic anhydride to be injected at high temperature of GC-MS.

MS approach allows the measurement of the mass isotopomer distributions (MID). The MID is the number of the relative amount of each mass isotopomer for each measured metabolite. As previously described, isotopomers are molecules that differ only in the number of isotopes incorporated, defined as M+0, M+1, M+2, M+*n* (where *n* is the number of ^13^C atoms). MS methods enable to measure the relative amount of each mass isotopomer for each detected metabolite. Another advantage for applying GC-MS methods on MFA is the wide spectral database of metabolites that strongly support their identification by GC-MS analysis.

Yuan *et al.* introduced a new strategy based on GC-combustion-isotope ratio MS (GC-C-IRMS) to determine *in vivo* flux central metabolism of *C. glutamicum* involving 4 labelling degrees from 0.5% to 10% [1-^13^C] glucose mixed with naturally labelled glucose.^41^ Authors demonstrated that using GC-C-IRMS technique is possible to develop the experiments with extremely low amount of the expensive labelled substrates, such as 1% which is enough to measure the ^13^C enrichment in proteinogenic amino acid hydrolysates. This work was of great interest for industry because the laboratory scale fermentation of *C. glutamicum* (very important for amino acids production) has been translated into a large scale of bioreactors used in biotechnological processes that otherwise would have been much more expensive considering the cost of using 99% [1-^13^C] glucose. They also proved that conventional GC-MS methods of the experiment in the same conditions are not reliable due to the poor sensitivity of the instrument and to low amount of ^13^C tracers' enrichments in amino acids (0.001–0.05 atom percent excess-APE).^91^

Another promising technique to overcome limits of MFA-based GC-MS is the two-dimensional GC coupled to time-of-flight MS (GCxGCTOF-MS) applied on a unicellular alga *Chlamydomonas reinhardtii*, widely studied as model system in photo-synthesis.^36^ In addition to high resolution and high sensitivity, other advantages of this method are better separation of co-eluted peaks and the presence of second retention time/index for detected metabolites. Experiments were performed using ^13^C sodium hydrogen carbonate and ^13^C sodium acetate for carbon flow under photoautotrophic and mixotrophic growth. Authors identified several metabolites such as alanine, aspartic acid, leucine, lysine, anthranilic acid, fructose, glucose, fumaric acid, malonic acid, and glycerol. According to different pathways of ^13^C incorporation, data showed that the assimilation of carbon in photoautotrophic conditions proceeds *via* Calvin–Benson cycle while in mixotrophic conditions mainly by glyoxylic acid cycle and subsequent TCA cycle.

Recently, Long and Antoniewicz published a very detailed protocol for ^13^C-MFA using *E. coli* as case of study.^43^ They explained clearly how to measure isotopic labelling of amino acids from hydrolysed biomass proteins, glucose from glycogen and ribose from RNA by GC-MS. They used GC system to measure mass isotopomer distributions with DB-5 ms capillary column, connected to a single quadrupole MS operating at electron impact (EI) ionisation at 70 eV. MID can be easily calculated by the relative abundances of each successive mass isotopomer (*e.g.*, M+0, M+1, M+2, *etc.*…).

GC-tandem MS has been used as advanced analytical technique for isotopic analyses showing more advantages compared to full scan analysis. Using tandem MS more information can be obtained on positional labelling compounds because individual mass isotopomers (parent ion) can be isolated from the rest of the sample, trapped and then fragmented into smaller compounds (daughter fragments).^92^ Jeffrey *et al.* tested for the first time in 2002 the feasibility of mass isotopomer analysis using GC-tandem MS to determine pathway flux using ^13^C-enriched substrates.^40^ They compared the obtained results to those obtained by ^13^C NMR spectroscopy. Authors analysed glutamate and citric acid cycles from rat hearts perfused with three substrate mixtures: glucose plus [2-^13^C] acetate, [1-^13^C] glucose plus U/L insulin, and glucose plus [3-^13^C] pyruvate. They showed that although analysis by NMR was simpler, tandem MS could combine higher sensitivity and larger amount of information allowing better metabolic flux analysis, while full-scan MS was the least accurate method. Since tandem MS is based on more fragmentation steps, the key advantage is the detailed positional labelling information obtained by the daughter fragments.^96^ This was demonstrated for the first time in 2012 by Choi *et al.* who developed a method for measuring the complete isotopomer distribution of aspartate (all 16 isotopomers) using GC tandem MS (MS/MS).^93^ At this time, the major limitation of tandem MS was the lack of computationally efficient method for simulating isotopic labelling data. Then in 2015 Tepper and Shlomi presented a novel approach called tandemer (from tandem mass-isotopomer) for efficiently simulating MS/MS measurements of metabolites in a metabolic network that showed how all tandemer distributions can be computed by sequentially solving linear balance equations for sets of tandemer distributions with increasing size.^94^ However, only in 2019 it was demonstrated that tandem MS data can be used for ^13^C-MFA calculations using the EMU framework, which is currently the most used mathematical approach for modelling isotopic labelling in ^13^C-MFA and is the core of several main software.^95^

#### 2.2.3 LC-MS

There are many instances where certain metabolites are incompatible for GC-MS analysis or require derivatisation, making LC-MS the preferred approach in such cases. In contrast to GC-MS, the electrospray ionisation used in LC-MS is a soft ionisation technique which reduces in source fragmentation. This is important because the soft ionisation gives the additional information of *m*/*z* of primary ions (molecular ion radicals, protonated or deprotonated molecules) that possess low internal energy to undergo further fragmentation. To achieve further fragmentations triple quadrupole or ion trap instruments are commonly employed. GC-MS has been mostly used to measure MIDs of proteinogenic amino acids, characterised by a slow turnover, good stability, and high abundance. On the other hand, metabolic intermediates, with fast turnover, reflect the instantaneous biochemistry of the cell also after perturbations.^97^ However, metabolic intermediates in the cell are less stable and less abundant than proteinogenic amino acids. Therefore, intermediate metabolites are often analysed by LC-MS, due to its higher sensitivity and softer ionisation.

van Winden *et al.* presented a ^13^C-MFA method based on rapid sampling and quenching of microorganisms from a continuous cultivation, followed by extraction and detection by LC-MS of free intracellular metabolites, mainly glycolytic intermediates, such as glucose-6-phosphate and phosphoenolpyruvate.^48^

LC-tandem MS (LC-MS/MS) is a highly sensitive and specific technique that allows detection of molecules in the femtomolar range.^7^ As previously mentioned, in MS/MS experiments the signal/noise ratio and the mass transitions between parent and fragment ions are drastically improved. Kiefer *et al.* developed a straightforward MS/MS approach investigating the correlation between the mass isotopomers of fragment and molecule ions.^98^ Authors applied this method to phosphorylated metabolites because their fragmentation results in high yields of [PO_3_]^−^ (*m*/*z* = 79) and/or [H_2_PO_4_]^−^ (*m*/*z* = 97) ions. They determined the mass isotopomer distribution from the molecule ions [M–H]^−^ obtained by multiple ion monitoring and from the carbon free fragment ions obtained by MRM. Using this method, a very small biomass amount is necessary for the analysis, allowing very small cultivation volumes in the microliter range.

In the same year, Iwatani *et al.* determined the metabolic flux changes during fed-batch cultivation from measurements of intracellular amino acids by LC-MS/MS.^49^ They applied this method to lysine production in fed-batch culture of *E. coli* and they compared this “new” approach with the conventional approach based on (i) continuous culture; (ii) assumption that MDV_free amino acids_ (mass distribution vector) is equal to MDV_proteinogenic amino acids_. For the first time, it was considered the isotopic effect of protein degradation on the intracellular metabolite pools, coming from the incorporation of the naturally labelled carbon from medium components into cellular metabolism. This approach made the basis for investigating cellular behaviour in industrial processes. Until the first decade of “2000s”, all LC-MS-based ^13^C-MFA studies investigated only ^13^C-data of intact molecules without positional information obtained by fragmentation. Then, Rühl *et al.* exploited for the first time collisional fragmentation of central carbon metabolites by LC-MS/MS.^50^ Authors acquired product ion scans for 20 intermediates of the central carbon metabolism to compile a catalogue of all possible fragments. Results showed more precise estimates of fluxes in pentose phosphate pathway, glycolysis, and tricarboxylic acid cycle.

Yuan *et al.* have used polarity switching selection reaction monitoring (SRM) mode on hybrid triple quadrupole MS coupled with hydrophilic interaction chromatography (HILIC) to monitor the incorporation of heavy atoms such as ^15^N and ^13^C.^99^ This approach has been reported to be robust, fast and reproducible for large-scale research and stable isotope labelled metabolites for clinical studies. There is an added benefit of improved sensitivity and dynamic range by using SRM over untargeted high-resolution LC-MS/MS. This approach is proving popular because method can be customised for specific pathways targeting isotope labelled metabolites using precursor and fragmentation ions by adjusting the mass to account for the labelled species. McCloskey *et al.* presented a novel LC-MS/MS method for measuring the precursor and product mass isotopomer distributions of metabolic intermediates and cofactors for MFA applications, the MID Max.^51^ This method has been applied on *E. coli* and has the advantage to maximize the number of mass isotopomer distributions that can be acquired in a single run. The authors also generated a library of product ion scans and spectral annotation that can be used for further experiments during the process. Recently, Yuan *et al.* presented a very detailed protocol for analysing incorporation of ^13^C and ^15^N into polar metabolites of central carbon metabolism and related pathways.^100^ This protocol is an extension of the protocol published in 2012 and it focused on targeted LC-MS/MS based approach using selected reaction monitoring (SRM) with polarity switching and amide HILIC to capture transitions for carbon and nitrogen incorporation into selected metabolites.^99^ The authors specified all details for reagent preparation and equipment setup, including the setting for the QTRAP mass spectrometer and HPLC system. The entire procedure presented by Yuan *et al.* (2019) takes 6–7 h for a single sample from experimental labelling and metabolite extraction to peak integration.

### 2.3 Computational modelling and data analysis software

Data collection and data analysis are the final steps to determine metabolic networks in organisms. Identification and quantification of isotopomers is a very delicate and laborious phase since this step requires the use of mathematical models to describe the relationship between isotopomer abundances and metabolic fluxes. Over the past decades various tools and approaches have been developed for mathematical modelling and computer simulation for metabolic system studies (Table 4).

**Table 4:** The main software used in computational modelling of biochemical networks

Software name	Web access	Ref.
Silicon cell	https://www.siliconcell.net	119
Systems Biology Markup Language (SBML)	https://sbml.org	120
JWS Online	https://jjj.bio.vu.nl	121 and 122
COPASI	https://copasi.org	123
CellNetAnalyzer	https://www2.mpi-magdeburg.mpg.de	124
BioNetS	https://x.amath.unc.edu/BioNetS	125
Fluxer	https://fluxer.umbc.edu	126

The ability to quantitatively describe metabolic fluxes through metabolic networks has been one of the major challenges of the early fluxomics *era*. The so called “model-driven” approach has been the most used method in the early stage of the systems biology when the sensitivity of mass spectrometry and NMR spectroscopy was not able to measure absolute concentrations of molecules with so much precision and accuracy as today.^82^ Usually, the first step of the “model-driven” approach is the generation of computational networks from existing knowledge of kinetics data and transcription rates (from scientific literature and/or lab-based), the second step is the integrative use of predictive models and the last step is the reduction of the known processes by selection techniques.^127^ Previously, most of the used methods required kinetic and concentration data of enzymes and cofactors but in the last two decades the most successful techniques such as constrain-based, genome-scale and FBA overcame this issue.^128^ For example, to deal with the lack of kinetic information, FBA based on optimality principles describes steady-state flux distributions in metabolic network using linear programming.^4,129,130^ In more detail, the first step in FBA is to mathematically represent metabolic reactions. The metabolic reactions are expressed as stoichiometric matrix (*S*) of size [*m* × *n*]. Every row of this matrix represents a metabolite (*m*), and every column represents a chemical or transport reaction (*n*). For each metabolite consumed there is a negative coefficient and *vice versa*, for each metabolite produced there is a positive coefficient, while the zero coefficient is used for those metabolites that do not participate in that reaction. The metabolites concentration is described by the following differential equation (*c*):
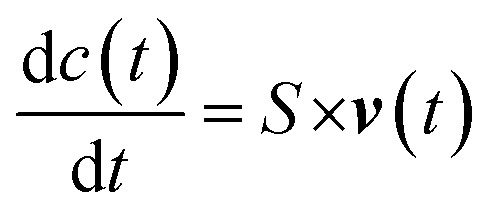
where ***v***(*t*) is the vector of reaction rates, including exchange fluxes that account for metabolites transport through the membrane. FBA assumes that metabolic dynamics have reached quasi- or pseudo-steady state, where metabolite concentrations do not change in time. In other word, the assumption is that each metabolite is consumed in the same quantity as it is produced, and this is expressed by the following equation:*S* × ***v***(*t*) = 0

This is also called “mass balance constraint” which explains that the *ex nihilo* formation of mass and even its vanishing is not possible. Additional constraints can be introduced in the model, such as the reversibility of reactions and compartments in which reactions take place. For example, upper and lower limits can be applied to individual fluxes (*v*_min_ ≤ *v* ≤ *v*_max_), for irreversible reactions *v*_min_ = 0. Because there are more reactions than metabolites (*n* > *m*), FBA does not have a unique solution for flux distribution but provides a “solution space” that contains all the possible steady-state flux distributions.^131^ To identify a single optimal flux distribution, it is necessary to state an objective function, which, in microbial cells, has generally been defined as the maximal cellular growth. Thus, in FBA the growth rate is maximised to predict metabolic fluxes. However, the assumption of optimality and steady state, and the application of a large set of constraints do not always reflect the actual cellular metabolism, thus the prediction is not always accurate. Moreover, the size and complexity of the huge dataset, composed by thousands of reactions (*e.g.*, 2251 reactions and 1136 metabolites were found in *E. coli* metabolism)^132^ requires a huge effort in computational modelling for their analysis. Therefore, a reduction procedure to obtain smaller models can sometimes be the best choice to study basic principle of organisms. Recently, Erdrich *et al.* presented the “NetworkReducer” which is a new algorithm to obtain smaller models using an automated reduction of metabolic reconstructions.^133^ The algorithm consists of two main steps: “pruning” and “compression”. Network pruning delivers a subnetwork of the system composed by a list of protected elements and functions (phenotypes) removing the inessential ones. This operation decreased the genome-scale network of *E. coli* from 2384 reactions to 455 and from 1669 metabolites to 438. Then, the compression further reduces the network size by collapsing some sequential reactions. The procedure has been applied to the iAF1260 genome-scale model of *E. coli* and in the end, they obtained 88 reactions and 69 metabolites.^133^

As for FBA also in ^13^C-MFA the metabolic reaction network can be reconstructed as a linear system where ***v*** = (*v*_1_, *v*_2_…, *v*_*n*_) is the flux vector and *S* is the stoichiometric matrix (*m* × *n*)*S* × ***v***(*t*) = 0

In this case, the “mass balance constraint” is valid for each isotopomer belonging to a metabolite. Another difference is that in ^13^C-MFA the metabolic fluxes are estimated from isotopic labelling data (simulated and measured *x*_s_ and *x*_m_, respectively) and extracellular rates measurements (simulated and measured *r*_s_ and *r*_m_, respectively), such as substrate uptake, oxygen uptake, growth and product secretion rates.^134^ Fluxes are estimated by iteratively minimising the sum of squared residuals (SSR) between the measured and simulated isotopic labeling patterns and external rate measurements^135^
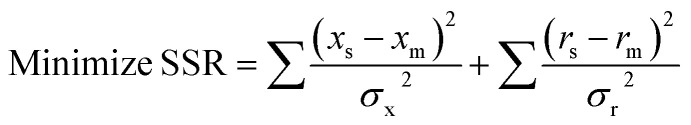


The goal is to find the set of fluxes that minimise the difference between observed and simulated isotope measurements. At each iteration, the isotopic labelling must be simulated for a set of fluxes by solving a sub-problem that consists of a large number of coupled non-linear equations.^134^ Several mathematical approaches have been developed to solve these non-linear isotope-labeling balances.^13,101,102,136^ The computational methods used for ^13^C-MFA have become progressively more advanced, especially by speeding up the calculations of metabolite labelling from an assumed set of fluxes. Schmidt *et al.* (1997) proposed an elegant method to reduce the model complexity of isotopomer models to that of classical metabolic models by expressing the 2^*n*^ isotopomer mass balances of a metabolite pool in a single matrix equation.^101^ The authors showed how the isotopomer model calculates the steady state label distribution in all metabolite pools as a function of the steady state fluxes.^101^ Wiechert *et al.* (1999) introduced the concept of cumomers, from “cumulated isotopomer fraction” referring to a certain sum of isotopomer fractions of a metabolite and provided an efficient procedure for solving isotopomer models.^102^ Then in 2007 Antoniewicz *et al.* developed a novel approach to simplify isotopomer calculations called the elementary metabolite units (EMUs) framework which is based on a highly efficient decomposition algorithm that identifies the minimal amount of labelling information required to simulate isotopomers of metabolites.^13^ The functional units generated by the decomposition algorithm, called EMUs, form the new basis for generating system equations that describe the relationship between fluxes and isotopomer abundances. The authors showed that the isotopomer abundances simulated using the EMU framework are identical to those obtained using the isotopomer and cumomer methods but the EMU models are orders-of-magnitude smaller than the isotopomer and cumomer models and require significantly less computation time.^13^ Many and different software tools are available to estimate metabolic fluxes, some of them are non-open source but available for academic research and some other are free for use (Table 5).^53,103,104^

**Table 5:** Selected commercial software to process metabolic flux analysis data

Software name	Compatible software	Techniques	Web access	Ref.
13C-FLUX2	MATLAB, Omix	LC-MS and NMR	https://www.13cflux.net/13cflux2/	105
INCA	MATLAB	GC-MS and NMR	https://mfa.vueinnovations.com/	106
METRAN	MATLAB	GC-MS	https://tlo.mit.edu/technologies/metran-software-13c-metabolic-flux-analysis	107
OpenFLUX2	Java, MATLAB	GC-MS	https://openflux.sourceforge.net/	108
OpenMebius	MATLAB	MS	https://www-shimizu.ist.osaka-u.ac.jp/hp/en/software/OpenMebius.html	109
SumoFlux	MATLAB, INCA	MS	https://gitlab.ethz.ch/z/sumoflux	45
tcaCALC & SIM		^13^C NMR, MS and tandem MS	https://www.invivometabolism.org/tca.html	87
VistaFlux	Omix	LC-MS	https://www.agilent.com/en/products/software-informatics/masshunter-suite/masshunter-for-life-science-research/vistaflux-software	110
WuFlux	MATLAB	GC-MS	https://www.13cmfa.org/	111
ScalaFlux	OpenMebius, INCA, R	MS, MS/MS, NMR	https://github.com/MetaSys-LISBP/IsoSim/	112
FluxML	Omix	NMR	https://www.13cflux.net/fluxml/validator/	113
FluxPyt	Python	MS	https://sourceforge.net/projects/fluxpyt/	114

Most of the software use the EMU framework to calculate isotopomers distribution. Among them, the most popular packages are OpenFLUX and 13C-Flux.^28,115^ OpenFLUX is a versatile and intuitive software package that permits to compile the EMU-based model from an Excel spreadsheet and generates MATLAB readable files allowing researchers to easily create models or experimental designs.^44^ The software is open source available upon request for Microsoft Windows XP, MacOS 10.4.11 and Ubuntu linux 7.1 operating systems but it requires the MATLAB Optimisation Toolbox. Additionally, it is possible to choose another algorithm package for flux estimation or alternative numerical approaches for flux analysis. OpenFLUX consists of two parts: submission of reaction data, in this step the investigator needs to choose the type of reaction network in the model definition file while in the second step the software will calculate cell fluxes as well as statistics using EMU framework. The authors developed an updated version, OpenFLUX2, with some more advantages compare to the previous version, such as calculation of parallel labelling experiments and fluxes measurement correlation, structural identifiability analysis and elements of experimental design.^108^ The OpenFLUX2 interface is similar to the previous version but has been updated with new features and it is more user-friendly (manual is available online).

13C-Flux is another option available for the detailed quantification of intracellular (quasi) steady-state fluxes.^28^ Recently, it has been updated to 13C-Flux2 with advanced features such as highly efficient implementation of EMU simulation algorithms, specification of arbitrary analytical technique (*e.g.* LC-MS/MS, ^13^C-NMR) and the FluxML, a standardized XML-based document format to specify all types of measurements.^105^ It consists of several steps: network modelling and measurement specification, simulation of isotope labelling states, parameter estimation, statistical quality analysis and experimental design (https://www.13cflux.net/13cflux2/). Results can be easily post-processed with MATLAB, moreover the software Omix, which is a network drawing tool, may be used for graphical and visualization purposes.^116^ This software is available with a commercial license and it has been developed and tested to run on Linux/Unix system, however, it is free of charge for universities and non-profit research institutes.

Zamboni *et al.* described a user-friendly software named FiatFlux for non-expert users who open their research window to metabolic flux analysis.^115^ FiatFlux was developed on a MATLAB basis, with programming language MATLAB R14 (The Mathworks) and allows to use the MATLAB Optimisation Toolbox. For non-expert users this software is relatively easy and more intuitive because it allows to calculate the flux ratios directly from MS data. In particular, it consists of two modules, the first calculates fluxes obtained from ^13^C tracers detected at GC-MS from amino acids, then automatically identifies fragmentation pattern and operates statistical analysis in the RATIO module, while the second module (NETTO) allows to determine fluxes in and out of the cell and therefore absolute intracellular fluxes. Nowadays, FiatFlux is almost entirely replaced by SUMOFLUX a new method based on a machine learning predictor trained using *in silico*^13^C dataset comprising thousands of data between flux ratios and corresponding labelled patterns.^45^ This *in silico* dataset is practically used for training and for prediction of metabolic network. They validated the method estimating five key flux ratios of the central metabolism of *E. coli* and *Bacillus subtilis* by GC-MS of proteinogenic amino acids upon silylation. In addition, SUMOFLUX allows measurements data also from parallel experiments where different ^13^C tracers have been used.

The tcaSIM program is another predictive software primarily applied to MS and ^13^C NMR spectroscopy. The tcaSIM program simulates flow of carbon isotopic tracer atoms through the TCA cycle and connected networks and when used in combination with the tcaCALC (which is a MATLAB tool that facilitates the quantitative analysis of metabolic tracing experiments) performs isotopomer analysis to estimate pathway fluxes in biological systems at metabolic and isotopic steady state.^117^

Recently, a new software called METRAN has been developed as a freely available tool for academic and educational use (https://tlo.mit.edu/technologies/metran-software-13c-metabolic-flux-analysis). A request should be sent through email to obtain a copy (mranton@udel.edu). METRAN allows to calculate MID for ^13^C-MFA and works in correlation with MATLAB language. Yoo *et al.* used this software for quantifying the reductive carboxylation flux from glutamine to lipid in adipocyte cells and they compared the result to that obtained from another software, ISA.^34^ The authors found that both software ISA and METRAN provided similar results, but ISA model requires only the MID of a single molecule as input, while METRAN models can use MID of all metabolic intermediates and fragments of selected pathways. Therefore, METRAN provides much more information about metabolic flux analysis. To estimate fluxes, METRAN minimises the variance between the observed and predicted measurements using non-linear least-squares regression approaches. The user needs to communicate to software how many and which tracers are using and with which ratios they should be mixed. An example of metabolic network for *E. coli* and its data set is distributed with METRAN software and analysed following a detailed protocol based on MID.^43^

In addition to previous software, the Isotopomer Network Compartmental Analysis (INCA) is the first software package that can perform both steady-state metabolic flux analysis and isotopically non-stationary metabolic flux analysis, meaning that this software simulates transients CLE also if ^13^C measurements are acquired before the isotopic steady state, as well as OpenMebius.^106,109^ Data extrapolated from INCA can be analysed and modelled with MATLAB. The software relies on EMU and network models extrapolated from it are compatible with other MFA software, such as OpenFLUX. Another important characteristic of INCA is that allows multiple and simultaneous experiments (replicates or parallel experiments) to generate a single flux map. The INCA application is free of charge for academic users. However, the software only supports MID data and proton NMR fractional enrichment data while the ability to specify for ^13^C-NMR fine spectra and tandem MS/MS data will be update in the future versions. For LC-MS analysis there are other software available, for example VistaFlux allows flux visualization and pathway integration for Agilent MassHunter LC-MS data. This software can create target lists, extract batch isotopologues, and visualize stable isotope label flux results on pathways for MS-only data from Agilent TOF-based high resolution LC/MS systems.^118^ Although some studies have highlighted limited performance in quantitative predictions of stoichiometry-based approaches,^137^ recently it was developed a two-step computational pipeline incorporating raw ^13^C-isotopic labelling data under multiple genetic and environmental perturbations and the computationally inexpensive parameterisation algorithm K-FIT^138^ demonstrating the potential use of a single metabolic network for both flux elucidation and kinetic parameterisation.^139^

In case no known algebraic relationship exists between isotopic labelling data and physiologically relevant variables, multivariate regression models, such as multiple linear regression (MLR), partial least-square regression (PLS) and regression using artificial neural networks (ANN), have been proposed for the analysis of isotopomer flux data to estimate physiological parameters for metabolism of mammalian gluconeogenesis.^140^

Interestingly, a completely different approach from the abovementioned computational models is based on highly simplified macro-kinetic representations of metabolism aiming to study emergent properties such as biomass growth and the production of specific metabolites. This type of approach has been mainly used in the field of industrial chemistry and applied microbiology.^141–143^ Among these, Mazzoleni *et al.*^144^ following the principles of System Dynamics,^145^ presented a first model simulating the growth of two strains of *Saccharomyces cerevisiae* (CEN.PK113-7D and CEN.PK2-1C) in batch and fed-batch cultures. More recently, the same model was extended to two prokaryotic species, *E. coli* and *B. subtilis*.^146^ In this paper, the authors explicitly stated that this modelling procedure directly aims at the identification of the minimal number of processes sufficient to simulate the emergent properties of a complex system based on logical reasoning and existing knowledge of the same system. Instead of considering the whole range of reactions and then reduce them, this modelling approach starts with the most basic mathematical representation of a very limited number of processes and increase the complexity by adding only those processes that are necessary to reach a representation of the system behaviour consistent with the aim of the specific model. In other words, the peculiarity of this different approach is that rather than reducing the number of reactions from thousands to hundreds, it starts selecting and fixing the main variables in order to obtain a minimal number of equations able to reach a significant fitting with observed data.^146,147^ In particular, this model was based only on 7 ordinary differential equations representing the main steps of glucose metabolism, *i.e.*, glucose uptake from the medium, glycolysis (from glucose-6-phosphate to pyruvate), respiration and/or ethanol/acetate production by fermentation, reserves accumulation and production of growth-associated inhibitory by-products.^146,147^ Taking into account only these selected (and compressed) processes, without explicit representation of any secondary pathway, the model is able to provide a robust representation of cell growth under all tested feeding conditions.

### 2.4 Application areas

^13^C-MFA played a crucial role in furthering the basic understanding of metabolism in general of different species.^38,148^ For example, ^13^C-MFA was applied to evaluate CHO cell metabolism, experiments suggest that promoting oxidative TCA cycle and PPP flux may provide a possible strategy to increase specific antibody production and reduce lactate accumulation during the production phase of industrial fed-batch CHO cell cultures.^149^ The use of ^13^C labelling experiments also helped to detect a significant transketolase-like protein 1 flux in CHO cells at the stationary phase and in 2017 has been published the first direct evidence that circulating lactate contributes to central metabolism in human non-small-cell lung cancers *in vivo*.^150,151^ Considering *E. coli* metabolism important contributions were given using a metabolic flux ratio (METAFoR) analysis and CLE that revealed how the central carbon metabolism of *Escherichia coli* responds to genetic and environmental manipulations.^39,152,153^

In the last three decades the “omics” sciences have seen an exponential growth in different fields, including agriculture, industrial processes, and medicine. Nowadays, metabolic engineering is one of the main areas for fluxomics application. The modification of specific biochemical reactions results in improvement of cellular properties under different environmental conditions.^154^ This economically and environmentally friendly technology appeals many industrial processes, which have as main goal the increase of specific metabolites production.^155^ For example, the demand for the essential amino acids, such as l-lysine, is rapidly growing in the global market, for their wide use as feed additive, composition of pharmaceuticals, cosmetics, and polymer materials. Xu *et al.* engineered the carbohydrate metabolism systems in *Corynebacterium glutamicum* to enhance the efficiency of l-lysine production from mixed sugar.^156^ Firstly, they engineered a functional metabolic pathway of sucrose and fructose through introduction of fructokinase from *Clostridium acetobutylicum*, then they further increase the l-lysine production by replacing the phosphoenolpyruvate-dependent glucose and fructose uptake system by inositol permeases and ATP-dependent glucokinase. Results showed that the engineered *C. glutamicum* produces 221.3 ± 17.6 g L^−1^l-lysine with productivity of 5.53 g L^−1^ h^−1^ and carbon yield of 0.71 g g^−1^ glucose in fed-batch fermentation.

Cyanobacteria are prokaryotic organisms that grow very fast and they can perform oxygenic photosynthesis starting from water, sunlight, and CO_2_. These characteristics make them the target organisms of metabolic engineering for photosynthetic production of biofuels. Knoot *et al.* recently presented an overview about MFA applied on the metabolic pathways that have been engineered in cyanobacteria to increase the production of several chemicals such as ethanol, isobutanol, isopropanol, isoprene and isoprenoids, succinate, and glycerol.^157^ Authors listed in their study most of the bioproduction strategies from carbon dioxide that have been adopted in different cyanobacterial strains.

^13^C-MFA was applied in metabolic engineering also to maximise the overall production yield and productivity of acetol, which is mainly used as an organic intermediate to produce polyols and acrolein and as a reducing agent in the textile industry. Nowadays, the chemical processes for acetol production are petroleum based. However, Yao *et al.* applied ^13^C-MFA to identify bottlenecks in the bioconversion of glycerol into acetol by *E. coli.*^15^ The overexpression of nadK encoding NAD kinase or pntAB encoding membrane-bound transhydrogenase resulted in increasing the acetol titer from 0.91 g L^−1^ to 2.81 g L^−1^. In another study on metabolic engineering of *E. coli* strains with ^13^C-metabolic flux analysis, authors increased the isopropyl alcohol yield of 55% mol mol^−1^ glucose by combining nitrogen-starved culture conditions with metabolic redirection from glycolytic flux to the glucose 6-phosphate dehydrogenase (G6PDH) and Entner–Doudoroff (ED) pathway.^158^

Another important example of the MFA application in the metabolic engineering field is represented by the bioproduction of riboflavin, mainly used as food and feed additive but also as food colorant and pharmaceutical. The constant increase demand of this vitamin from the world market driven the research community to move its production from chemical synthesis to biotechnology strategies.^159^ Schwechheimer *et al.* resolved carbon fluxes of a complex industrial process of riboflavin biosynthesis, using ^13^C isotope experiments, vegetable oil as raw material and the fungus *Ashbya gossypii* as main producer.^160^ The rational process optimisation resulted in a final riboflavin titer increase of 45%.

Regarding the biomedical field, ^13^C-MFA plays an important role to elucidate the complex mechanism of toxicity^161^ and diseases,^16^ but also to better understand the effect of drugs and to unravel novel drug targets.^162,163^ Dong *et al.* proved that isotopic labelling and network analysis are very powerful tools for identifying the main metabolic pathways (*e.g.*, reductive metabolism of glutamine, altered glycolysis, serine, acetate and glycine metabolism, the isocitrate dehydrogenase mutations) in cancer cells.^164^ It has been demonstrated that metabolic disfunction is an essential hallmark of tumorigenesis, and MFA provides suitable framework to study human disease.^165^ Interestingly, the study of metabolic fluxes in human lung cells has already seen application for inhibiting the reproduction of SARS-CoV-2 through perturbations of metabolic network.^166^ Although the authors developed a computational approach, the predicted model can offer a platform for rapid formulation of drugs experimentally testable (through MFA) against this emerging virus responsible of the world pandemic started in 2019.

## 3 Conclusion

Metabolic flux analysis has emerged as a powerful technique for providing important quantitative information of biological systems otherwise unobtainable from other “omics” approaches. Based on well-known stoichiometry from scientific literature and experimental data, metabolic pathways of biological systems can be predicted in advanced and ^13^C-MFA can be applied to quantify actual cellular fluxes in prokaryotic organisms. New and non-invasive methods allow quantification of *in vivo* fluxes. The use of stable isotopes as ^13^C in MFA is becoming extremely important in the study of metabolic network having as main applications areas metabolic engineering, biomedical and pharmaceutical research. The development of analytical techniques such as mass spectrometry and NMR spectroscopy and the use of dedicated software tools highly increased the precision and the accuracy of flux analysis. New and increasingly sophisticated computational methods have found application in systems biology. Moreover, the use of a modelling procedure inspired by macro-kinetic models developed in the field of applied microbiology and based on a minimal number of processes to simulate the emergent properties of a biological system, should be explored in ^13^C-MFA. Indeed, further progress in metabolic flux analysis greatly depends on the improvement and co-application of analytical methods, software packages and optimised computational modelling.

## Funding

This work was supported by the Green Chemicals Beacon of Excellence, University of Nottingham.

## Conflicts of interest

There are no conflicts to declare.

## Supplementary Material
